# Imputing Missing Data in Hourly Traffic Counts

**DOI:** 10.3390/s22249876

**Published:** 2022-12-15

**Authors:** Muhammad Awais Shafique

**Affiliations:** 1 Centre Internacional de Mètodes Numèrics en Enginyeria (CIMNE), Universitat Politècnica de Catalunya BarcelonaTech (UPC), 08034 Barcelona, Spain; ashafique@cimne.upc.edu; Tel.: +34-93-401-74-95; 2 Center for Innovation in Transport (CENIT), Universitat Politècnica de Catalunya BarcelonaTech (UPC), 08034 Barcelona, Spain; 3 Department of Civil Engineering, University of Central Punjab, Lahore 54590, Pakistan

**Keywords:** AADT, ATR, daily volumes, imputation, missForest, missing data

## Abstract

Hourly traffic volumes, collected by automatic traffic recorders (ATRs), are of paramount importance since they are used to calculate average annual daily traffic (AADT) and design hourly volume (DHV). Hence, it is necessary to ensure the quality of the collected data. Unfortunately, ATRs malfunction occasionally, resulting in missing data, as well as unreliable counts. This naturally has an impact on the accuracy of the key parameters derived from the hourly counts. This study aims to solve this problem. ATR data from New South Wales, Australia was screened for irregularities and invalid entries. A total of 25% of the reliable data was randomly selected to test thirteen different imputation methods. Two scenarios for data omission, i.e., 25% and 100%, were analyzed. Results indicated that missForest outperformed other imputation methods; hence, it was used to impute the actual missing data to complete the dataset. AADT values were calculated from both original counts before imputation and completed counts after imputation. AADT values from imputed data were slightly higher. The average daily volumes when plotted validated the quality of imputed data, as the annual trends demonstrated a relatively better fit.

## 1. Introduction

The hourly traffic volume is an essential dataset to be collected and maintained for understanding the operational characteristics of a highway network. This dataset leads to the calculation of average annual daily traffic (AADT), a parameter that is of utmost importance in transportation infrastructure analysis and design. Furthermore, the current hourly volumes can be used to predict future traffic so that designers can anticipate the traffic conditions and design accordingly. The hourly volume data also provide insight into the seasonal variations and patterns during special events, such as important games, implementation of key regulations, and extreme weather events.

AADT is used to calculate the design hourly volume, which in turn is used in the design of roadway capacity and level-of-service (LOS) analysis. The designer cannot use the highest hourly volume recorded for a particular road type, as it would be a wasteful use of resources. Hence, designers go for a small compromise, where demand is allowed to exceed the capacity for some predefined number of hours in the year. For this purpose, AADT is multiplied by a K-factor, which signifies the proportion of AADT occurring during the peak hour. The typical range of K-factors is from 0.08 to 0.10 on urban freeways and from 0.09 to 0.13 on rural freeways [1]. Hence, it is evident that for proper infrastructure design, accurate calculation of AADT is essential, for which correct and complete hourly traffic volumes need to be collected.

Continuous hourly traffic volumes can be collected using several methods, most notably by using automatic traffic recorders (ATRs). The ATRs are installed permanently at strategically located points in the road network. In addition to permanent stations, temporary stations can also be set up to collect data for a short duration. Various technologies are used in the ATR, such as pneumatic tube counters, magnetic counters, inductive loops, radar devices, and surveillance cameras. Depending on the data collection requirements, the type of ATR device can be selected. Real-time data from all count stations are received and archived by a traffic data center for internal and external use.

Missing data in hourly traffic volumes can result in incorrect AADT calculation, leading to inefficient transportation infrastructure design. Correctly imputing the missing data would allow the closing of this error. Several studies have worked on achieving this goal. A study examined the data imputation practices followed by traffic agencies in west Canada [2]. It was reported that Saskatchewan and Manitoba used the previous year’s data from the same station to replace missing values. Further, the statistics contractor responsible for collecting and managing data for Alberta was found to use historical data for monthly average daily traffic estimation.

Another study conducted in the United States revealed that 13 states would use some procedures to replace missing values when temporary devices failed [3]. However, 23 states used some data imputation procedures when permanent devices malfunctioned [4]. In this regard, several different methods were used. For instance, in Alabama, the previous year’s data was used for imputation if less than 6 h were missing. The day was discarded if more than 6 h were missing. A straight-line estimate using data from months before and after the malfunction was employed for imputation in Delaware. Data from the same day of the week of the same month was used in Oklahoma to fill in the missing values. Various imputation methods followed by traffic agencies were compared in a study using ATR data from Alberta and Saskatchewan [5]. It was reported that methods taking into account additional information and utilizing sophisticated prediction models performed much better. Another research aimed at imputing traffic count data during the holiday season claimed the superior performance of k-nearest neighbor (KNN) [6].

A study compared imputation methods, such as mean, median, expectation maximization (EM), multivariate imputation by chained equations (MICE), k-nearest neighbor (KNN), and random forest (RF), to tackle the problem of missing values in hourly traffic volume data [7]. Data imputation by median value was determined to be the best method. However, the accuracy of imputation was not evaluated as an independent entity; rather, the accuracy recorded was for hourly volume prediction for a later year. Therefore, imputations performed by the different methods may be limited by the prediction capacity of the algorithms used i.e., recurrent neural network (RNN), long short-term memory (LSTM), and gated recurrent unit (GRU).

Imputation is mostly performed by extracting candidate values from neighboring data records that have similar information to the data record containing missing values. These candidate values may sometimes contradict each other, so a study developed a constraint mechanism to address this issue, where some tolerances were introduced in the similarity rule [8]. In another unique study, the authors developed an imputation model for traffic congestion level data (CIM) based on joint matrix factorization [9]. Firstly, a tensor of order-3 containing the traffic congestion data was constructed. Repetition of data and resemblance among roads was then modeled using location-based and time-based information. Later, constraints were applied to ensure the results were consistent across time. The results demonstrated that by utilizing the characteristics associated with congestion patterns, the missing data could be imputed at a higher accuracy than several of the other methods studied.

Using non-motorized count data from several cities in Oregon, a study investigated several different imputation methods [10]. It was concluded that random forest performed the best but was difficult to apply. On the other hand, the day-of-year (DOY) factor method was simple to apply and worked well when the number of missing values was significant. For low amounts of missing values, negative binomial regression showed good results.

A data imputation structure, named HoloClean, based on probabilistic inference, was developed by a group of researchers to link the existing numerical data repairing methods with the comparative approaches [11]. Another study compared the data repairing accuracy of HoloClean with their developed method based on relaxed functional dependencies, which yielded better results [12].

The objective of this study is to use hourly traffic volumes collected by ATR and compare the recent approaches used for data imputation. Traffic count data from ATR in New South Wales (NSW), Australia is cleaned and then explored for missing data. Both levels of missing data, i.e., hourly values missing within available days and entire days missing, are analyzed. Although it is an acceptable practice to omit unreliable and missing days from AADT calculation, this study explores the possibility of imputing those complete days as well. This aspect also serves as a unique contribution of this study. The improvement in AADT calculation is demonstrated in the end.

The structure of this article is as follows: Section 2 describes the data used in this study, as well as the data cleaning performed. Section 3 presents the various data imputation methodologies applied in this article. In Section 4, the analysis and preliminary results comparing the several imputation methods used are provided. Section 5 covers the imputation of actual missing data and the subsequent calculation of AADT. Section 6 provides additional discussion, followed by conclusions and future work in Section 7.

## 2. Data

The data used in this study was collected by Roads and Maritime Services, New South Wales (NSW), Australia. Roads and Maritime Services continuously collect traffic volume counts, speed, and classification using nearly 600 permanent roadside collection device stations. In addition to permanent stations, sample roadside collection device stations are also set up across NSW to collect data on a short-term basis.

To ensure the accuracy of the data for the annual average daily traffic (AADT) calculation, the agency recommends that it should pass the following three checks.

- At least 19 hourly observations are required per day.
- At least one value for each day of the week is required per month.
- The daily volume should be within 20% of the average for that day of the week in the month.

### 2.1. Data Selection

Despite being designed to collect traffic volume data continuously, the collection devices could display varying unreactive periods. Hence, the collection agency defined the collected data in terms of data availability and data reliability. Data availability refers to the percentage of available data within the recorded duration, whereas data reliability indicates the percentage of usable data within the available data.

**Figure 1:**
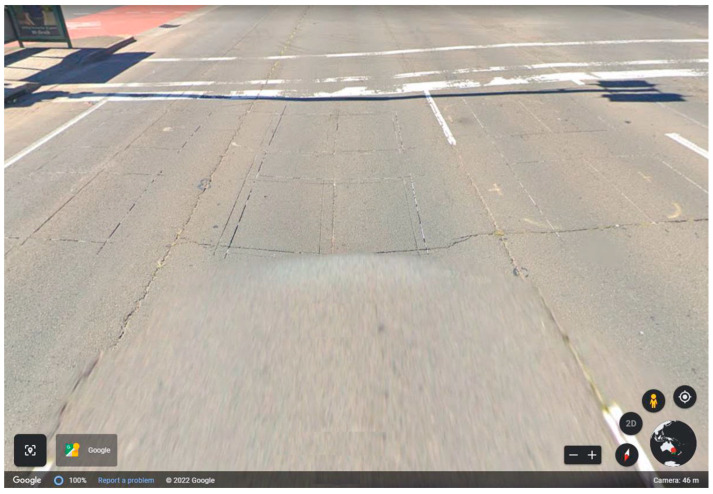
Google Street View of loop induction device.

**Figure 2:**
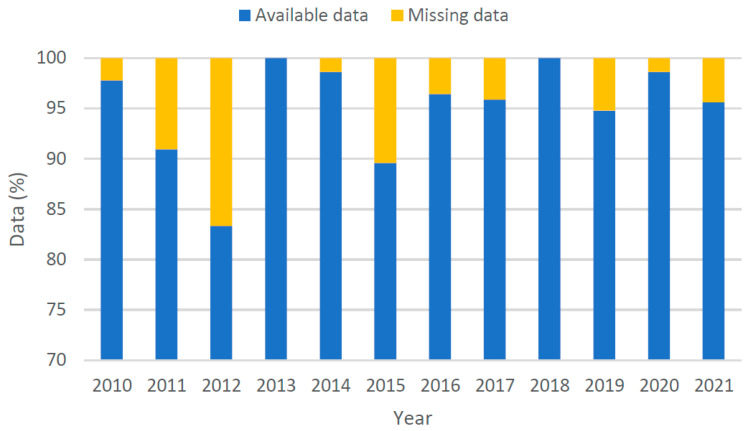
Available traffic count data for each year.

To understand the volume data more closely and to determine the best course of action for imputing the missing data, one data collection station was picked out. The selected station (station key 56820) was chosen because it contained the maximum number of years where the data availability and data reliability were higher than 95%. Only light vehicle data in the direction of the counter was considered. The selected station consisted of an Excel loop induction device, as shown in Figure 1. It is present on Centenary Drive, east of Richmond Road in Sydney. Classified as an arterial road, the drive consists of three lanes in each direction. Figure 2 shows the status of data available for each year.

### 2.2. Data Cleaning

**Figure 3:**
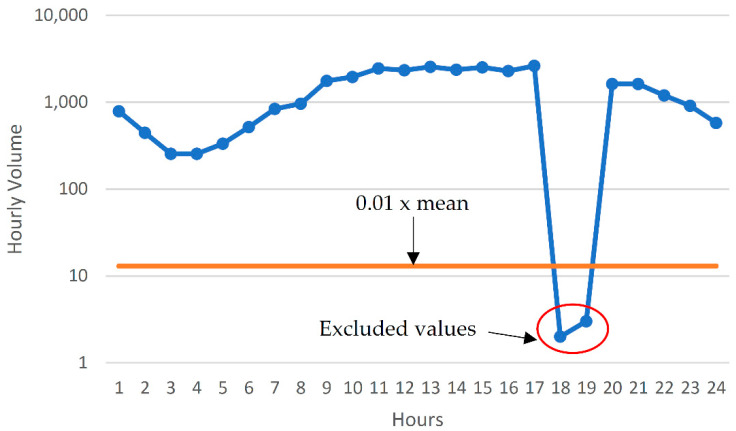
Identifying and excluding unacceptably low values.

**Figure 4:**
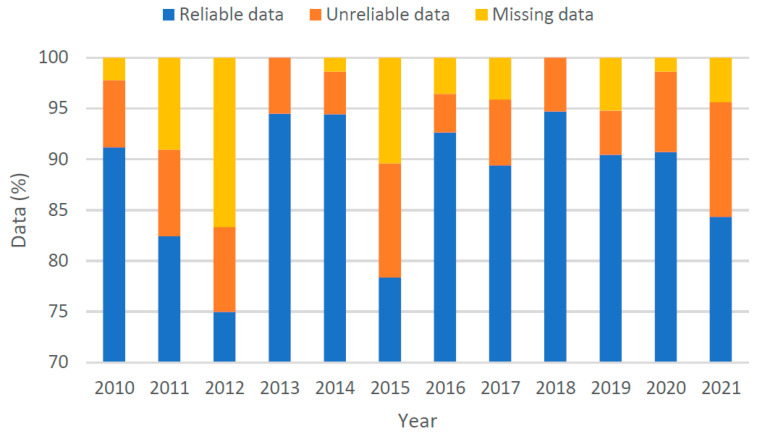
Reliable traffic count data after cleaning.

The available data, as shown in Figure 2, was screened for irregularities. Firstly, all daily volumes for a particular day of the week in a particular month were checked to ensure that they lay within 20% of their mean value. This resulted in the identification of some days having inconsistent volumes. The data revealed numerous hourly volumes to be missing (replaced by NA). It was also observed that some inconsistent hourly volumes were recorded, such as an hourly volume of 2 recorded while the mean hourly volume for that day was above 1000. Consequently, a high-pass filter was implemented such that all hourly volumes less than 1% of the mean hourly volume for that day were replaced with “NA”, as demonstrated by data from just one such day in Figure 3. Hence, not only complete days were missing, but hourly volumes from within available days were also missing. The distribution of data after cleaning is shown in Figure 4.

## 3. Data Imputation Methodologies

Although there are a huge number of algorithms used for missing data imputation present in literature, this study took into account three types, owing to their frequent use specifically for imputing missing values in hourly traffic volume data. The three classes used were: (1) multivariate imputation by chained equations (MICE), (2) random forest (RF), and (3) extreme gradient boosting (XGBoost). Each of them is briefly described in the following sections.

### 3.1. Multivariate Imputation by Chained Equations

Multivariate imputation by chained equations (MICE) uses Gibbs sampling to generate multiple imputations for multivariate missing data. There is no compulsion as to where the missing data may be located within the given dataset. Based on the other data columns, the algorithm generates reasonably fabricated values to impute an incomplete column. Each incomplete column (target column) has its own unique set of predictors. (By default, it consists of all the columns except the target column.) Any incomplete predictors are essentially imputed before being used to impute the target column.

The imputation methods used within the framework of MICE are discussed below.

#### 3.1.1. Predictive Mean Matching

Predictive mean matching (PMM) imputes the missing data by selecting real values from the data. For each missing value, the imputation method picks out a small set of complete cases where the predicted value is closest to the one for the case containing that missing value. From this set of candidate values, one value is randomly selected to impute for the missing value. The imputation method used is based on [13].

#### 3.1.2. Weighted Predictive Mean Matching

Being mostly similar to the previous method, bootstrapped sampling is used as the initial step for weighted predictive mean matching. The methodology is based on [14,15]. The procedure involves extracting a bootstrap sample from the parent data. Using the leave-one-out method, a beta matrix is estimated for the bootstrapped sample. Type II predicted values are calculated for the observed cases and the missing cases. Distances are then calculated between all observed values and their linking missing values. These distances are converted to probabilities of drawing candidate values. Based on these probabilities, a donor is drawn for each missing value. The observed value associated with the donor is then used to replace the missing value.

#### 3.1.3. Random Sample from Observed Values

From the entire dataset, a random value is extracted to replace the missing value. This is a very simple imputation method that might not yield the best results. No criterion is followed to ensure the selected value follows the general trend of the data.

#### 3.1.4. CART within MICE

Classification and regression trees (CART) can be used from within the framework of MICE. To start, the missing values are initially replaced by random values extracted from the observed values related to each missing variable. The first variable containing at least one missing value is marked as the target variable, while all the remaining variables become predictors or features. Taking into account only the cases with observed values, a tree is fitted using the data. The resulting tree holds a subset of observed values on each of its leaves. The first missing value is fed to the tree, ending at one of the leaves. A value is randomly taken from the subset of observed values on that particular leaf and used for imputation. The process is repeated for all the variables containing missing values. Each complete cycle covering all incomplete variables is repeated several times to finally get one dataset of imputations. Multiple imputation datasets are yielded when the complete procedure is repeated many times.

#### 3.1.5. Random Forest within MICE

Similar to CART, random forest can also be applied for data imputation from within the framework of MICE. Initially, k number of bootstrapped samples are drawn from the complete dataset. One tree is grown for each of the bootstrapped samples. Each tree is then used to determine the imputations, similar to the procedure followed by CART. Apart from applying random forest imputation from within MICE, it was also included in the comparison as a separate algorithm.

#### 3.1.6. Unconditional Mean Imputation

This imputation method calculates the mean of the variables and replaces the missing values for that variable with the calculated mean. Since the mean value remains the same, any additional iterations would be unnecessary. A drawback of this method is that it disregards any relationship present between variables, hence resulting in a loss of correlation.

#### 3.1.7. Bayesian Linear Regression

Based on the linear connection between the parameters and conforming to the normal distribution, this method is used to very efficiently determine the most appropriate replacements for the missing values. The posterior distribution of model parameters is used to draw the parameters. Predictions for the missing values are generated from a Gaussian distribution, with a mean equal to the product of the weight matrix (transpose) and the predictor matrix and the variance equal to the square of the standard deviation.

#### 3.1.8. LASSO Linear Regression

Least absolute shrinkage and selection operator (LASSO) can be applied to linear regression. Data is shrunk towards a central value, for instance, the mean. The imputation method is based on the direct use of regularized regression (DURR) [16,17]. For any missing variable, a bootstrapped sample is drawn, with replacement, from the original dataset. The observed values for that particular variable are taken as the target class, while the remaining corresponding variables are treated as the predictors. A regularized regression model is fitted on this observed data. Using the posterior distribution of the model parameters, a vector of randomly drawn regression coefficients is obtained. The predictive distribution is defined by the original dataset, not the bootstrapped dataset, and is used to impute the missing value.

#### 3.1.9. LASSO Select + Linear Regression

This is a combination of the previous two methods. LASSO variable selection is used as a preprocessing step that leads to the application of Bayesian linear regression. The imputation method is based on the indirect use of regularized regression (IURR) [16,17]. Using the complete cases in the dataset, a regularized linear regression model is fitted, and the active set of predictors is identified. The posterior distribution is estimated by applying a standard inference model, such as maximum likelihood. Utilizing the posterior distribution, values from the predictive distribution are randomly drawn to impute for the missing data.

#### 3.1.10. Random Indicator for Non-Ignorable Data

A method that iterates over the response and assignment models is used in the random indicator method to estimate a balance between the distribution of the missing data and the observed distribution. The predictors of the response model and the imputation model are assumed to be identical in this procedure.

### 3.2. Random Forest

Random forest can be grown and used to impute missing data. Imputation can be performed by regression or by missForest. The latter has been shown to outperform many of the popular data imputation methods [18]. The missing values are initially filled by the mean of the respective column (continuous data) or by the statistical mode (categorical data). The dataset is then divided into training and testing datasets. The training data consists of all the observed cases, whereas the testing data contains the cases with missing values. Random forest is trained and then used to make predictions for the missing values. One iteration cycle is concluded once all the missing values are imputed. Several iterations are performed, controlled by a stopping criterion, to get the final imputation values.

### 3.3. Extreme Gradient Boosting

Extreme gradient boosting (XGBoost) can also be used to use bootstrapping and predictive mean matching for the imputation of missing data [19]. When used under fully conditional specification (FCS), XGBoost imputation models are developed for each incomplete parameter. At the start, the variables in the data are sorted according to the number of missing values. An initial imputation is performed to fill in the missing values. XGBoost is then used to make predictions for the missing values. PMM is used to extract a set of donors closely associated with the predicted value. A donor case is randomly selected, and its observed value is used to impute the missing value.

The suitable values of hyperparameters, as determined by grid search, were: gamma = 2, eta = 0.1, max. depth = 18, and min. child weight = 0.6.

## 4. Analysis and Results

### 4.1. Evaluation Measures

Two types of accuracies were calculated based on mean absolute error (MAE) and root mean squared error (RMSE), as given by the following equations.

(1) $$\mathrm{Error},\;e=\mathrm{Imputed}\;\mathrm{value}\;\left(I\right)-\mathrm{Actual}\;\mathrm{value}\;\left(A\right)$$

(2) $$\mathrm{MAE}=\frac{1}{n}\sum \left|e\right|$$

(3) $$\mathrm{MAE}\%=\frac{\frac{1}{n}\sum \left|e\right|}{\frac{1}{n}\sum A}=\frac{\sum \left|e\right|}{\sum A}$$

(4) $$\mathrm{RMSE}=\sqrt{\frac{1}{n}\sum e^{2}}$$

(5) $$\mathrm{RMSE}\%=\frac{\sqrt{\frac{1}{n}\sum e^{2}}}{\frac{1}{n}\sum A}$$

(6) $$\mathrm{Accuracy}\;\left(\mathrm{MAE}\right)=100-\mathrm{MAE}\%$$

(7) $$\mathrm{Accuracy}\;\left(\mathrm{RMSE}\right)=100-\mathrm{RMSE}\%$$

### 4.2. Analysis

To test the various imputation methods, the unreliable part of the data was removed. A total of 25% of the remaining data was randomly selected to form the test data. For any day to be included, at least 19 hourly observations are necessary; hence, the missing data can be either a maximum of 5 hourly observations in a day (21%) or a complete day (100%). Consequently, the testing was performed for two scenarios. In the first case, 6 hourly observations (25%) for each testing day were randomly selected and replaced with “NA”. For the second case, the entire testing data (100%) was replaced with “NA”. This not only provided the opportunity to compare the imputation methods but also to assess if different methods were suitable for the two scenarios.

### 4.3. Results

**Figure 5:**
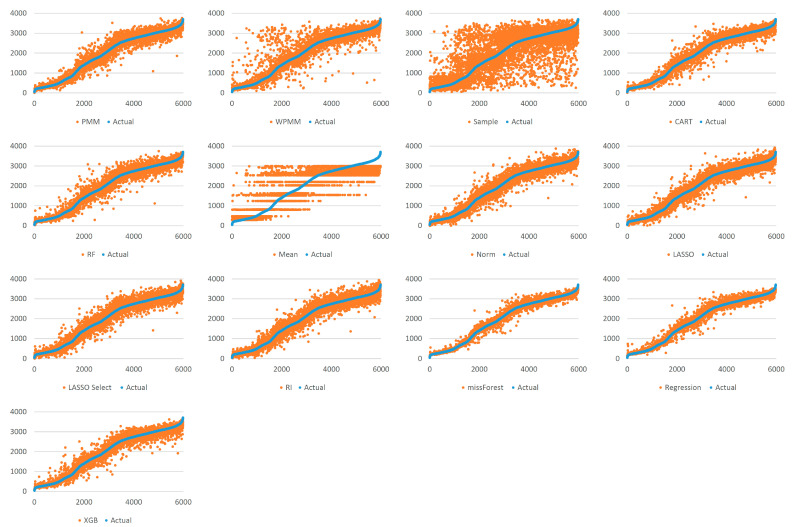
Spread of predictions for 25% missing data.

**Figure 6:**
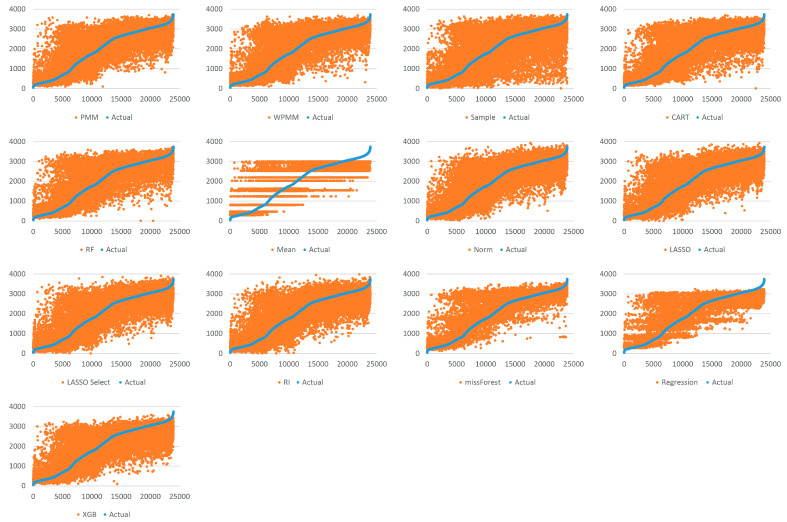
Spread of predictions for 100% missing data.

**Figure 7:**
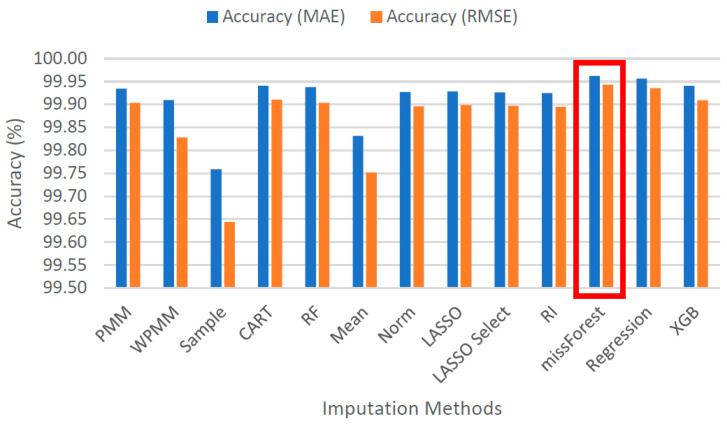
Imputation accuracies for 25% missing data.

**Figure 8:**
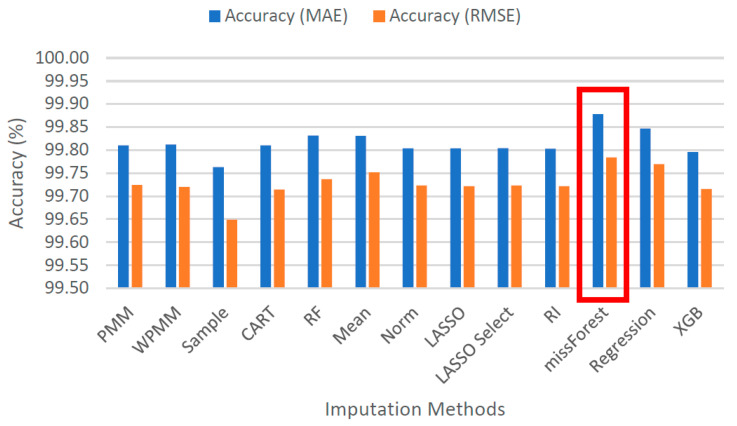
Imputation accuracies for 100% missing data.

Figure 5 demonstrates the spread of predicted values by each method for 25% missing data. Each graph shows the actual values sorted in ascending order and their respective predicted values as obtained using several methods. It is evident that taking random samples for data imputation results in a considerable deviation from the true values. The second worst case can be observed for mean value imputation. Another observation is that predictive mean matching works better than weighted predictive mean matching. All the other methods show a mostly similar extent of spread, with missForest showing the most compact picture in the case of 25% missing data. For 100% missing data, similar graphs are provided in Figure 6. Due to the increased complexity of this task, the figure shows a significant deviation from the actual values for all methods. It is difficult to identify the best-performing algorithm, although missForest shows a slightly compact trend. To investigate further, imputation accuracies were calculated, as shown in Figure 7 and Figure 8. It is evident from the figures that missForest outperforms all other methods in both testing scenarios, closely followed by the random forest regression method. Furthermore, the accuracy achieved for 100% missing data by missForest is slightly lower than that achieved for 25% missing data, with an accuracy drop of 0.08% in terms of MAE and 0.16% in terms of RMSE. One can also deduce that although missForest provides the highest accuracy, the accuracy differences with other methods are not very substantial. Hence, other methods can also be utilized with a slight decrease in imputation accuracy.

Once missForest was confirmed to be the relatively more suitable imputation methodology, further investigation was performed to find the optimum values for associated hyperparameters, which are provided in Table 1. Two important observations can be made here. Firstly, different values would be better for both testing scenarios, and secondly, complete day imputation (100%) would require multivariate missForest, whereas partial day imputation (25%) would benefit from missForest.

## 5. Data Imputation and AADT Calculation

**Figure 9:**
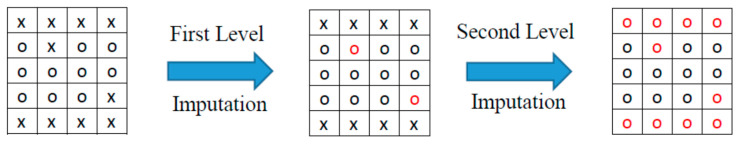
Data imputation method.

Next, the actual missing data and the unreliable data were imputed in two levels, as demonstrated in Figure 9. In the first level, missing hourly values within available data were imputed using missForest. This completed data was then used to impute the missing days using multivariate missForest in the second level.

**Figure 10:**
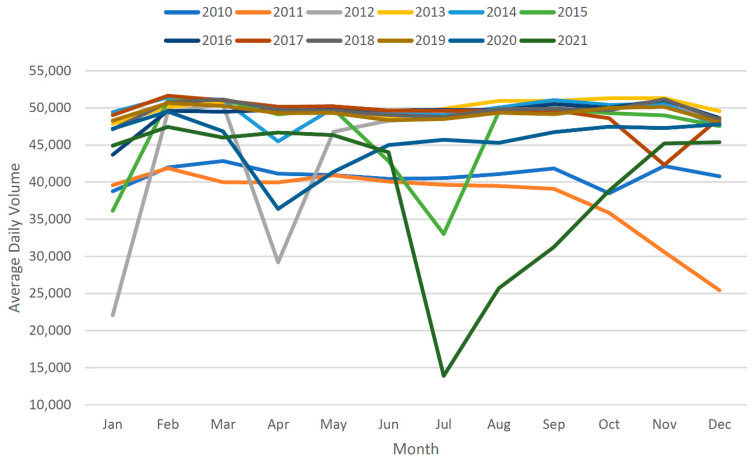
Average daily volume trends before imputation.

**Figure 11:**
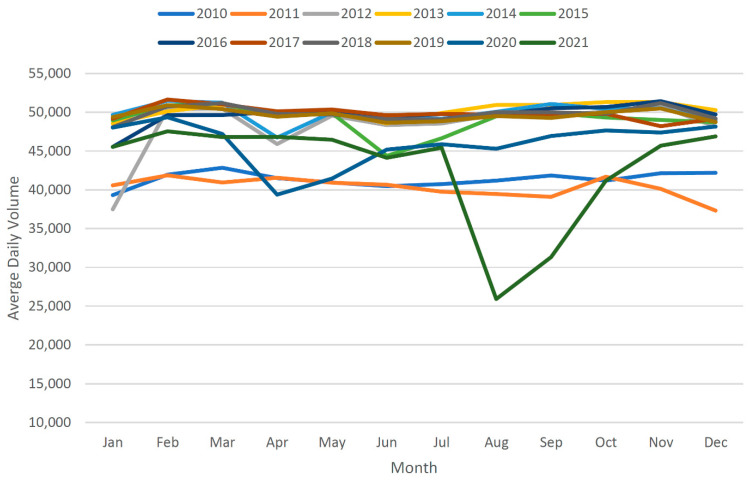
Average daily volume trends after imputation.

Table 2 gives the AADT calculations for before and after data imputation. It can be observed that AADT values differ more in years when there are more missing. Moreover, the completion of data with the assistance of imputation always results in a relatively higher AADT value. The effectiveness of data completion can be checked by plotting the average daily volumes before and after imputation, as performed in Figure 10 and Figure 11. It is evident from Figure 10 that the original data demonstrate substantial variations, whereas the traffic should have shown relatively similar trends over the years. However, the plot derived from imputed data in Figure 11 shows relatively better traffic patterns.

## 6. Discussion

The analysis and the results documented in this study reveal that data imputation using missForest and multivariate missForest can result in not only complete hourly data but also the general trends of the data captured, resulting in better accuracy with the replacement of missing data. The sudden dip in the data for the year 2021, as demonstrated in Figure 9, is due to the characteristics of the reliable data used. If the reliable data statistics are relatively lower or higher than the usual trend, then the same will be carried forward, even after data imputation.

Since it is an acceptable approach to exclude problematic days from the calculation of AADT, the agency applying the imputation can go for level 1 imputation only. However, when using the current hourly volumes to predict future hourly volumes, it would be beneficial to complete the dataset by applying the second-level imputation as well.

Another observation made after the analysis is that AADT values calculated after imputation are always slightly higher than the original values. Further, the difference in AADT is relatively higher for years that have a higher proportion of missing data. This may mean that imputation results in modest overestimation. However, this cannot be concluded as certain and will require further investigation in the future.

## 7. Conclusions and Future Work

This study concludes that missing hourly volumes within available data can be accurately imputed by applying missForest, whereas data for complete missing days can be significantly replaced by employing multivariate missForest. This conclusion is derived after comparing the imputation accuracies of several methods mostly used for this purpose. It is further deduced that AADT calculated with the imputed data is more precise, since the average daily volumes calculated over the months show a better conformation to the general traffic volume trend.

There remain several unanswered questions. Will these conclusions apply to data from other ATR stations? Is it worthwhile to impute complete days, or should they be skipped while calculating AADT? What will be the impact of partial data on forecasting future hourly traffic volumes? Will forecasting be improved by using the completed dataset after imputation? All these questions will be investigated and appropriate answers found in future works.

## Figures and Tables

**Table 1:** Suitable values of hyperparameters.

Missing %	Number of Variables	Number of Trees	Tolerance
25%	1	400	0.01
100%	0.59	400	0.0001

**Table 2:** AADT calculations.

Year	Original		Imputed		AADT		% Diff
	Annual Volume	Days Recorded	Annual Volume	Days Recorded	Original	Imputed	
2010	13,657,299	333	15,093,344	365	41,013	41,352	0.826
2011	11,755,237	307	14,714,824	365	38,291	40,315	5.286
2012	13,076,838	281	17,652,549	366	46,537	48,231	3.641
2013	17,303,743	345	18,344,154	365	50,156	50,258	0.204
2014	17,156,610	345	18,202,197	365	49,729	49,869	0.281
2015	13,867,853	287	17,873,202	365	48,320	48,968	1.340
2016	16,821,151	341	18,177,959	366	49,329	49,667	0.685
2017	16,249,321	328	18,194,399	365	49,541	49,848	0.620
2018	17,218,272	347	18,182,104	365	49,620	49,814	0.390
2019	16,366,929	332	18,109,640	365	49,298	49,615	0.644
2020	15,191,121	333	16,830,915	366	45,619	45,986	0.805
2021	12,828,239	309	15,613,213	365	41,515	42,776	3.036

## Data Availability

All data used during the study are available online https://data.nsw.gov.au/search/dataset/ds-nsw-ckan-5facd2f4-0f5c-4637-ab17-36cdc1c0747b/details?q=traffic (accessed on 22 August 2022).
